# A schizophrenia study based on multi-frequency dynamic functional connectivity analysis of fMRI

**DOI:** 10.3389/fnhum.2023.1164685

**Published:** 2023-05-12

**Authors:** Yuhu Shi, Zehao Shen, Weiming Zeng, Sizhe Luo, Lili Zhou, Nizhuan Wang

**Affiliations:** ^1^College of Information Engineering, Shanghai Maritime University, Shanghai, China; ^2^Surgery Department of Tongji University Affiliated Yangpu Central Hospital, Shanghai, China; ^3^School of Biomedical Engineering, ShanghaiTech University, Shanghai, China

**Keywords:** functional magnetic resonance imaging, multi-frequency bands, dynamic functional connectivity, support vector machine, schizophrenia

## Abstract

At present, fMRI studies mainly focus on the entire low-frequency band (0. 01–0.08 Hz). However, the neuronal activity is dynamic, and different frequency bands may contain different information. Therefore, a novel multi-frequency-based dynamic functional connectivity (dFC) analysis method was proposed in this study, which was then applied to a schizophrenia study. First, three frequency bands (Conventional: 0.01–0.08 Hz, Slow-5: 0.0111–0.0302 Hz, and Slow-4: 0.0302–0.0820 Hz) were obtained using Fast Fourier Transform. Next, the fractional amplitude of low-frequency fluctuations was used to identify abnormal regions of interest (ROIs) of schizophrenia, and dFC among these abnormal ROIs was implemented by the sliding time window method at four window-widths. Finally, recursive feature elimination was employed to select features, and the support vector machine was applied for the classification of patients with schizophrenia and healthy controls. The experimental results showed that the proposed multi-frequency method (Combined: Slow-5 and Slow-4) had a better classification performance compared with the conventional method at shorter sliding window-widths. In conclusion, our results revealed that the dFCs among the abnormal ROIs varied at different frequency bands and the efficiency of combining multiple features from different frequency bands can improve classification performance. Therefore, it would be a promising approach for identifying brain alterations in schizophrenia.

## 1. Introduction

Blood oxygen level-dependent (BOLD)-based functional magnetic resonance imaging (fMRI) is a non-invasive brain function research method, which is characterized by high temporal resolution and spatial resolution. For example, Biswal et al. (1995) first extracted and analyzed the low-frequency range (<0.1 Hz) of BOLD signals, and they investigated the interaction among distributed brain regions using low-frequency fluctuations of BOLD signals. In particular, the reason why Biswal et al. chose the frequency range of 0.01–0.08 Hz was that they found there were almost no fluctuations above 0.08 Hz, and fluctuation was the largest at 0.01 Hz in the frequency domain.

Most of the rs-fMRI studies for the analysis and diagnosis of various psychiatric disorders focused on the entire low-frequency band (0.01–0.08 Hz) because it is believed to be related to neural oscillations (Biswal et al., 1995; Lowe et al., 1998). However, the human brain is a complex dynamic system spontaneously generating a multitude of oscillatory waves. For instance, some fMRI studies have revealed specific relationships between distributed neural activity and frequency bands (Meda et al., 2016; Braun et al., 2018), and the neuronal excitability is larger during a certain phase of the low-frequency band (Penttonen and Buzsáki, 2003). Frequency-dependent effects were found in different brain regions (Gohel and Biswal, 2015), brain networks (Esposito et al., 2013), functional hubs (Wang et al., 2018), and global signals (Wang et al., 2020). Thus, some studies are beginning to investigate the fMRI data in multi-frequency bands rather than conventional frequency bands. With the purpose of gaining insights into the neuropathogenesis behind many psychiatric disorders including schizophrenia, we aimed to investigate the hypothesis of whether the multi-frequency analysis can reveal more neuropathological information than the conventional frequency band. Thus, it may provide a potential way to study biomarkers for schizophrenia identification.

With regard to the separation of frequency bands, we followed the linear theory of natural logarithm (N3L), which was first proposed by Penttonen and Buzsáki (2003). They suggested that an independent frequency band was generated by distinct oscillators with specific properties and physiological functions (Penttonen and Buzsáki, 2003; Buzsáki and Draguhn, 2004). In this study, we followed the N3L and referred to the code provided in the multi-frequency analysis software for fMRI data named DREAM (https://github.com/zuoxinian/CCS/tree/master/H3/DREAM) (Gong et al., 2020). Thus, the fMRI signals were decomposed into several bands, including Slow-6 (0.0040–0.0111 Hz), Slow-5 (0.0111–0.0302 Hz), Slow-4 (0.0302–0.0820 Hz), Slow-3 (0.0820–0.2231 Hz), and Slow-2 (0.2231–0.25 Hz), according to the N3L. However, the fMRI signals <0.01 Hz were reported to be associated with scanner-related noise. Slow-6, Slow-3, and Slow-2 mainly reflect low-frequency drift, white matter signals, and high-frequency physiological noises, respectively (Zuo et al., 2010; Baria et al., 2011). Slow-5 and Slow-4 are investigated in this study as their combined frequency range is similar to the conventional frequency band.

Although there are only a few rs-fMRI studies for neurological diseases based on multi-frequency bands, they have achieved better diagnostic effects than studies based on conventional frequency bands. Most studies have adopted amplitude of low-frequency fluctuations (ALFF) analysis, which is a classical method in fMRI research and is widely used in the analyses of neurological diseases (Yang et al., 2007). The study of Zuo et al. proved that ALFF and fALFF have relatively high test–retest reliability (Xing and Zuo, 2018; Zuo X.-N. et al., 2019; Zuo X. N. et al., 2019). Through applying ALFF analysis to a multi-frequency scheme, Parkinson's disease (Tian et al., 2020) is believed to be more associated with the Slow-4 frequency band, while Alzheimer's disease (Hu et al., 2017), mild cognitive impairment (Han et al., 2011), depression (Wang et al., 2016), and other diseases are believed to be more associated with the Slow-5 frequency band. It was also found that Slow-4 and Slow-5 are more responsive to specific pathological information in schizophrenia (Hoptman et al., 2010; Yu et al., 2014).

Functional connectivity (FC) analysis is also an effective tool in clinical neuroscience research, which can reflect the relationship between neural activities in distributed brain regions, and it is an effective way to characterize the integration of brain functions (Friston, 2011). At present, the analyses of brain FC mainly include static functional connectivity (sFC) and dFC. Recently, ROI-wise sFC, as a classic method, has been widely used. The first step is using an atlas, such as Anatomical Automatic Labeling (AAL) (Tzourio-Mazoyer et al., 2002), to parcellate the human brain into a total of 116 regions. Then, N ROIs are selected to conduct a correlation analysis among the time series of each ROI, and a correlation matrix of N × N is obtained. The sFC assumes that the FC in the scanning period is a fixed value, which cannot reflect the dynamics of the internal information interaction in the brain (Calhoun et al., 2014).

To further explore the state of brain FC, researchers began estimating time-varying FC, known as dFC, on timescales ranging from seconds to minutes (Chen et al., 2017; Yang et al., 2022). dFC can not only observe the variation of the connectivity strength between different pairs of ROIs over time but also capture the spontaneously and repeatedly generated FC patterns (Gu et al., 2020). The sliding window correlation (SWC) technique is the commonly used method to study dFC (Handwerker et al., 2012; Allen et al., 2014). Many studies have found that brain diseases are associated with abnormal resting-state brain connectivity, and such abnormal resting-state brain connectivity associated with specific diseases can be further used as neural markers for the diagnosis and prediction of diseases (Nomi et al., 2016; Liu et al., 2017). However, the dFC analysis method is rarely used in multi-frequency band studies, but it also achieved good diagnostic and prediction performance (Luo et al., 2020). For example, Kottaram et al. (2018) adopted a dFC analysis based on sliding window correlation to predict schizophrenia and achieved high accuracy.

The machine-learning approach has recently attracted more and more attention in the investigation of brain connectivity (Rashid et al., 2016; Khatri and Kwon, 2022). In order to yield a higher accuracy rate, a more stable classifier, and investigate the most discriminated features, an FS method is required (Guyon et al., 2002; Rakotomamonjy, 2003). The recursive feature elimination with 10-fold cross-validation (RFECV) was employed to select features, and the support vector machine (SVM) optimized by a 10-fold cross-validation grid search was applied for the classification of schizophrenia patients and healthy controls. Furthermore, the fALFF analysis was performed to determine the findings for abnormal ROIs, while the dFC analysis was carried out to investigate the correlation characteristics among the abnormal ROIs from a time-varying perspective.

Therefore, we proposed a novel multi-frequency-based dFC analysis method and applied it to a schizophrenia study. The AAL atlas was adopted to parcellate the cortex into 90 ROIs, and three frequency bands (Conventional: 0.01–0.08 Hz, Slow-5: 0.0111–0.0302 Hz, and Slow-4: 0.0302–0.0820 Hz) were considered. Then, fALFF analysis was used to identify brain regions of interest, followed by dFC analysis between them. Finally, the classification performance was compared between the Combined frequency bands (Slow-4 and Slow-5) and the Conventional frequency band.

## 2. Materials and methods

### 2.1. Participants

All the data were collected from the public database OpenfMRI (https://openfmri.org/dataset/ds000030/). This study was supported by the Consortium for Neuropsychiatric Phenomics, and its accession number is ds000030 (Gorgolewski et al., 2017). After receiving a thorough explanation, participants gave written informed consent following procedures approved by the Institutional Review Boards at UCLA and the Los Angeles County Department of Mental Health. Demographic information and clinical characteristics of the participants were presented as follows: healthy Caucasian (non-Hispanic or Latino) adults, with ages ranging from 21 to 50 years, from the Los Angeles area, who had passed neuropsychological tests and had eight or more years of education.

The following participants were excluded to ensure dependable research results: people with nervous system disease, head trauma with loss of consciousness or cognitive sequelae, people who consumed drugs over the past 6 months, people with a mental history of serious mental illness or attention deficit hyperactivity disorder, major medical history, MRI contraindications (including pregnancy), scan on any mood change medications, and those who were left-handed. Finally, this study selected all 50 patients with schizophrenia (12 women, 38 men, average age ± SD: 36.46 years ± 8.79 years) and some matched healthy individuals. After the head motion correction step of data preprocessing, four patients and several controls were excluded. Finally, there were 46 patients with schizophrenia (12 women, 34 men, average age ± SD: 36.46 years ± 8.81 years) and 46 healthy individuals (12 women, 34 men, average age ± SD: 35.87 years ± 7.88 years).

### 2.2. Data acquisition

All neuroimaging data were obtained using a 3T Siemens Trio scanner. In the resting scan, participants were asked to remain relaxed and keep their eyes open for 5 min. They were protected from any stimuli throughout the scan. Functional images were obtained with a T2^*^-weighted echoplanar imaging sequence with the following parameters: time points = 152, slice thickness = 4 mm, slice number = 34, TR = 2,000 ms, TE = 30 ms, flip angle = 90°, matrix = 64 × 64, and FOV = 192 mm. The duration was 304 s, and each subject produced 152 images. A T1-weighted high-resolution anatomical scan was acquired with the following parameters: slice thickness = 1 mm, slice number = 176, TR = 1,900 ms, TE = 2.26 ms, flip angle = 7°, matrix = 256 × 256, and FOV = 250 mm.

### 2.3. Data preprocessing

The resting-state fMRI raw data were preprocessed referring to DPABIV5.1 (http://rfmri.org/dpabi) (Yan and Zang, 2010), which integrates SPM12 (https://www.fil.ion.ucl.ac.uk/spm) and REST (http://www.restfmri.net). The preprocessing using DPABIV5.1 includes the following eight steps: (1) The first 10 time points were removed; (2) slice timing correction was performed; (3) head motion correction was performed, and the participants with head movement over 1.5 mm and rotation over 1.5 degrees were removed; (4) T1 structure images were registered to functional image; (5) the images were divided into gray matter, white matter, and cerebrospinal fluid by Diffeomorphic Anatomical Registration Through Exponentiated Lie Algebra (DARTEL) (Ashburner, 2007); (6) noise signals such as linear drift, white matter, and cerebrospinal fluid were removed. Friston 24-parameter model was selected to adjust head movement parameters, and SPM prior template was used to remove global signals (Fox et al., 2005, 2006); (7) The images were registered to Montreal Neurological Institute templates, and the image voxels were resampled to 3 × 3 × 3 mm^3^; and (8) a 6 × 6 × 6 mm^3^ FWHM Gaussian kernel is used for spatial smoothing to reduce the registration error and increase the signal-to-noise ratio.

### 2.4. Methods

**Figure 1:**
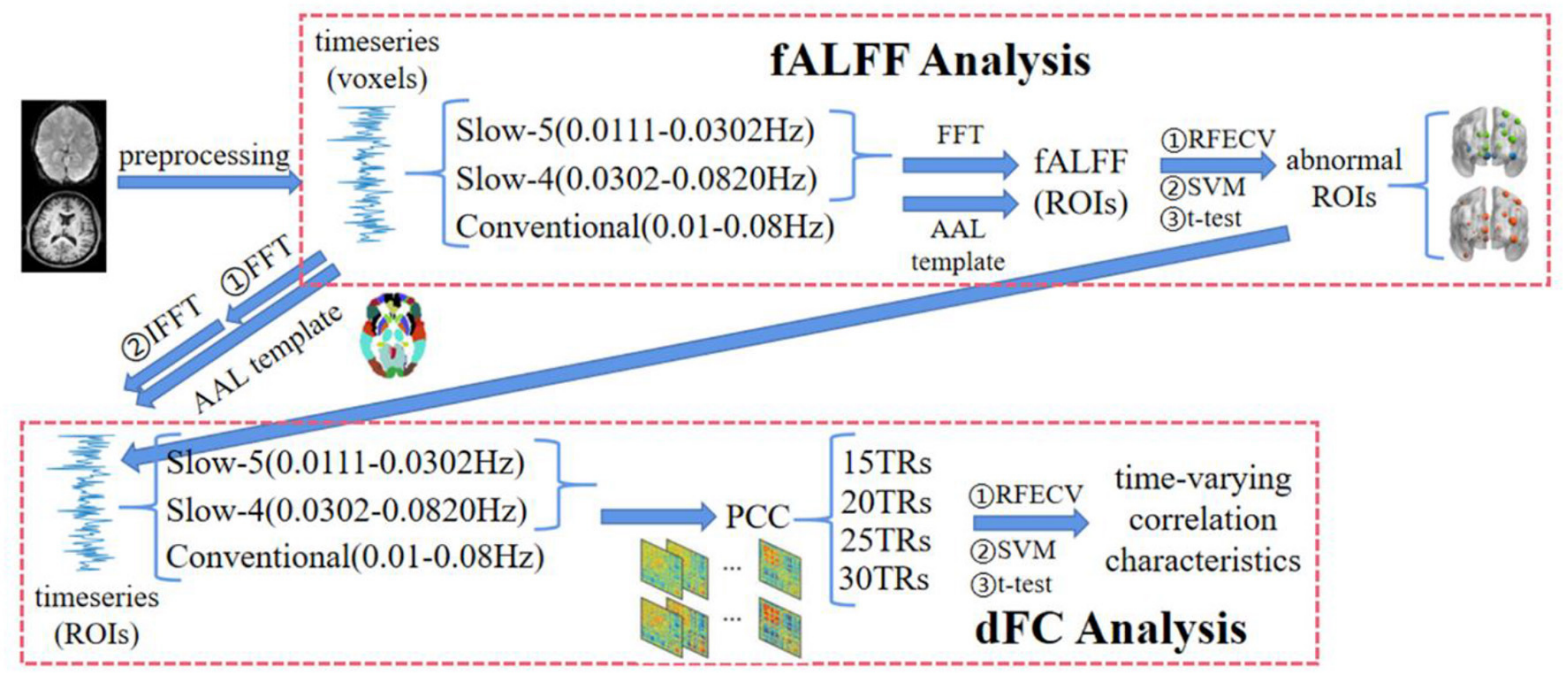
The flowchart of the multi-frequency-based dynamic FC analysis method, which consists of fALFF analysis and DFC analysis. Among them, the fALFF was used as the feature to select the brain regions with significant differences between SZ and HC in each frequency band by *t*-test and RFECV in the fALFF analysis. Then, the functional connections between these brain regions under different window-widths were calculated in the DFC analysis, and the functional connections with significant differences between SZ and HC were further obtained by *t*-test and RFECV. Finally, the selected features with the best classification performance using SVM were the time-varying correlation characteristics for the schizophrenia study.

The flowchart of this study is shown in Figure 1, including preprocessing, fALFF analysis, and dFC analysis.

#### 2.4.1. (Inverse) Fast Fourier Transform

Fast Fourier Transform (FFT) is an efficient method to calculate the Discrete Fourier Transform. It was constructed by Cooley and Tukey (1965) according to the periodicity and symmetry of the twiddle factor. For the simple polynomial multiplication problem, the most direct multiplication method needs Θ(*n*^2^) time, while the FFT algorithm needs Θ(*nlgn*) time to complete the evaluation and interpolation operation by using the special property of complex unit roots.

The Discrete Fourier Transform formula for calculating the signal vector x is presented as follows:


(1) $$\begin{array}{l} X\left(k\right)=DFT\left[x\left(n\right)\right]=\overset{N-1}{\underset{n=0}{\sum }}x\left(n\right)W_{N}^{nk},0\leq k\leq N-1 \end{array}$$

The corresponding Inverse FFT is presented as follows:


(2) $$\begin{array}{r} \;\;\;\;\;\;\;\;\;\;\;\;x(k)=IDFT[X(k)]=\frac{1}{N}\sum_{k=0}^{N-1}X(k)W_{N}^{-nk\;}\\ =\frac{1}{N}{\left[\sum_{k=0}^{N-1}X^{n}(k)W_{N}^{nk}\right]}^{n}=\frac{1}{N}{\left\{DFT[X^{N}(k)]\right\}}^{n},0\leq k\leq N-1\;n \end{array}$$

where $W_{N}^{kn}$ is called the rotation factor and presented as follows:


(3) $$\begin{array}{l} W_{N}^{nk}=e^{-j\frac{2\pi }{N}nk} \end{array}$$

#### 2.4.2. Decomposition of the frequency band

Theoretically, the range of each frequency band is fixed, namely frequency intervals. If the TR interval is not fast enough or there are not enough time points, the precise value of the theoretical frequency range cannot be extracted. To address this issue, the time points were increased in this study, because the TR interval cannot be changed. Thus, zeros were padded to 2e12 after the time series similar to padding zeros after the time series when performing an FFT algorithm. Therefore, the frequency bands in this study have higher resolution, and we were able to precisely decompose the entire frequency of 0–0.25 Hz into the seven frequency bands.

With regard to the decomposition of the frequency band, we followed the N3L and referred to the code provided in DREAM (Gong et al., 2020). According to the parameter description of fMRI data involved in this study, each voxel has *N* = 142 time points at TR = 2 s interval. Hence, the maximal frequency we can reliably investigate is no more than 0.25 Hz [ = 1/(2 × TR)]. Furthermore, zeros are padded after the end of time points to improve frequency resolution and the number of time points is padded to 2e12. Thus, the lowest frequency can be conservatively estimated using equation 1/(*N* × TR/2) and equals 2.44e-04 Hz. Furthermore, the minimal reliable frequency equals 7.32e-04 Hz [= 0.25/(2e12 + 1)]. Finally, the fMRI signals can be divided into seven frequency bands, namely, Slow-8 (7.32e-04–0.0015), Slow-7 (0.0015–0.0040), Slow-6 (0.0040–0.0111 Hz), Slow-5 (0.0111–0.0302 Hz), Slow-4 (0.0302–0.0820 Hz), Slow-3 (0.0820–0.2231 Hz), and Slow-2 (0.2231–0.25 Hz).

**Figure 2:**
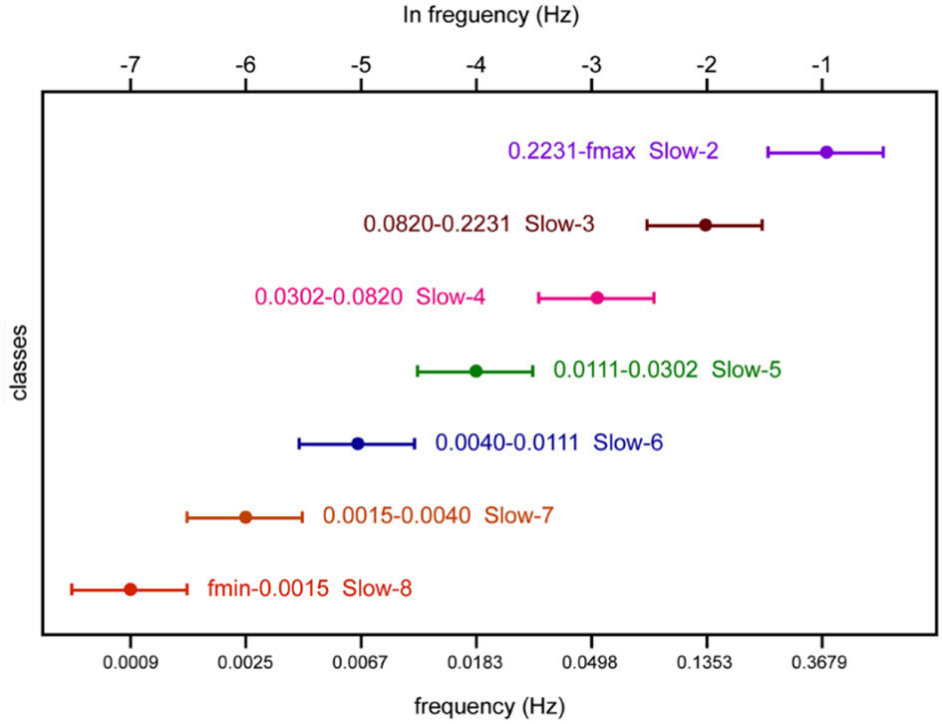
Schematic representation of oscillatory classes involved in this study. Frequency bands form an arithmetic progression on the logarithmic scale.

For each band, the range of frequencies is shown in Figure 2, together with its term. The ratio between neighboring bands is approximately equal to the natural logarithm e (Penttonen and Buzsáki, 2003). Meanwhile, an arithmetic progression on the logarithmic scale corresponds to a difference of 1. Thus, the frequency bands form an arithmetic progression on the logarithmic scale. For each band, the lower/upper limits were obtained by dividing/multiplying the center frequency by 1.65. Particularly, we could not get the upper limit in Slow-2 because the maximal frequency we could reliably investigate was no more than 0.25 Hz. Thus, the range of Slow-2 was 0.2231–0.25 Hz.

#### 2.4.3. Fractional amplitude of low-frequency fluctuation analysis

Analysis of low-frequency fluctuations was proposed by Zang et al. (2007), which can be used to describe the strength of local brain activities. However, there are some disadvantages, such as excessive noise and energy accumulation. To improve ALFF analysis, Zou et al. (2008) proposed fractional ALFF (fALFF), which can be regarded as normalized ALFF. In each frequency band, we performed fALFF analysis to identify those brain regions with discriminative fALFF in schizophrenia.

The specific method for calculating fALFF is described as follows: First, read in the preprocessed time series *x*(*t*) [in equation (4)]. Then, obtain the power spectral density by performing the FFT algorithm on time series *x*(*t*). After that, ALFF is calculated as the sum of amplitudes within a specific low frequency range [in equation (5)]. Finally, fALFF can be calculated by dividing ALFF across the entire frequency band to ALFF in the specific frequency band [in equation (6)].


(4) $$\begin{array}{lll} x\left(t\right) & = & \overset{N}{\underset{k=1}{\sum }}\left[a_{k}\cos\left(2\pi f_{k}t\right)+b_{k}\sin\left(2\pi f_{k}t\right)\right] \end{array}$$

(5) $$\begin{array}{lll} ALFF & = & \underset{k:f_{k}=band\;range}{\sum }\sqrt{\frac{a_{k}^{2}\left(f\right)+b_{k}^{2}\left(f\right)}{N}} \end{array}$$

(6) $$\begin{array}{lll} fALFF & = & \underset{k:f_{k}=band\;range}{\sum }\sqrt{\frac{a_{k}^{2}\left(f\right)+b_{k}^{2}\left(f\right)}{N}}/\overset{N}{\underset{k=1}{\sum }}\sqrt{\frac{a_{k}^{2}\left(f\right)+b_{k}^{2}\left(f\right)}{N}} \end{array}$$

In fALFF analysis, a total of 271,633 voxels (3 × 3 × 3 *mm*^3^ spatial resolution) were considered first. The fALFF per voxel was calculated by applying the function mentioned above on the preprocessed time series. Then, a matrix of subjects × voxels could be obtained from fALFF estimations in each of the three frequency bands (Conventional: 0.01–0.08 Hz; Slow-5: 0.0111–0.0302 Hz; Slow-4: 0.0302–0.0820 Hz). Next, the representative fALFF per ROI was calculated by averaging voxel-based time series within every ROI. Thus, the feature matrix from fALFF estimations at each band was subjects × ROIs. Practically, the Combined (Slow-4 and Slow-5) scheme had a feature of subjects × ROIs × 2 and the Conventional scheme had a feature of subjects × ROIs.

After applying feature selection and classification methods to the feature sets, abnormal ROIs would be found in the Combined and Conventional schemes, respectively. The selected features with the best classification performance were the abnormal ROIs and would be the targeted ROIs for dynamic functional connectivity analysis.

#### 2.4.5. Dynamic functional connectivity analysis

For the Combined scheme, the time series of the abnormal ROIs in Slow-5 and Slow-4 were extracted, respectively. Then, the time-varying characteristics in the two frequency bands were combined, which was the feature set for dFC analysis. For the Conventional scheme, the abnormal ROIs in Slow-4 and Slow-5 were utilized to extract their time series in the Conventional scheme. Then, two matrices of the time-varying characteristics were combined, which was the feature set for dFC analysis.

The sliding window correlation (SWC) technique is the most commonly used method for dFC analysis, which is reflected by measuring the FC of ROIs in each temporal segment. The formula for calculating the FC correlation coefficient matrix is as follows:


(7) $$\begin{array}{l} r^{t}\left(t\right)=corr\left(x_{t}^{t+w-1},y_{t}^{t+w-1}\right) \end{array}$$

where *r*^*t*^(*t*) represents the coefficient of the sliding window correlation between two groups of time series, *w* is the sliding window-width, $x_{t}^{t+w-1}$ and $y_{t}^{t+w-1}$ represent the time series from time *t* to *t*+*w*−1, and *corr*( ) refers to PCC.

Laumann et al. (2016) found that the correlation was relatively stable if they were calculated in a short period of time. The sliding window-width of 30–60 s achieved the best ability to identify the correlation characteristics and to reflect the cognitive state of people (Shirer et al., 2012). For example, Yu et al. (2015) obtained good research results at 20TRs sliding window-width analyzing fMRI data of schizophrenia patients.

In dFC analysis, four sliding window-widths (15 TRs, 20 TRs, 25 TRs, and 30 TRs) with five TRs step lengths were considered. For each kind of sliding window-width, the time series per voxel was calculated by applying equation (7) to the preprocessed time series. Then, the representative time series per ROI was calculated by averaging the voxel-based time series within every ROI. After that, correlation coefficients among the abnormal ROIs per temporal segment were calculated. Thus, a matrix of subjects × (temporal segments × abnormal ROIs × abnormal ROIs) can be obtained in a certain frequency band at a certain sliding window-width.

The results of the fALFF analyses were used to make decisions as to what regions should be used in the dynamic functional connectivity analyses. Combined and Conventional were used in both fALFF analyses and dFC analyses. In fALFF analyses, the abnormal regions were extracted using frequency domain signals. In dFC analyses, the abnormal functional connectivities were extracted using time domain signals.

#### 2.4.6. Feature selection and classification

An increasing number of fMRI studies are starting to use a machine-learning approach (Bae et al., 2018; Antonucci et al., 2019; Kumar et al., 2022). The recursive feature elimination with 10-fold cross-validation was employed to select features. The support vector machine tuned by a 10-fold cross-validated grid search was applied for classification.

The RFECV is a wrapper algorithm with two sections: recursive feature elimination and cross-validation. The specific steps of RFE in this study are as follows: (1) The initial feature set is all available features. (2) An external estimator, linear support vector regression, was given to assign weights (the coefficients of the estimator) to features. (3) Delete the feature with the lowest weight, then update the feature set. (4) Skip to step 2 until we have finished rating the importance of all features.

Cross-validation begins by dividing dataset D into K identical, disjoint subsets, each called a “fold,” and using one of them as a test set and the others as training sets in turn for each training session. In this way, it is equivalent to obtaining *K* groups of training sets and test sets, and the final prediction result is the average of *K* test results. Particularly, 10-fold cross-validation was performed to avoid problems caused by the improper partition of datasets (Shelatkar et al., 2022). Finally, we could obtain a robust rank of the features from most important to least important.

The RFE with 10-fold cross-validation was employed from the Scikit-learn toolkit to rank and weigh every feature according to its strength to distinguish between patients with schizophrenia and healthy controls. The *N* (1 ≤ *N* ≤ extracted features) ROIs with the highest weight were selected as features for classification.

SVM tuned by cross-validation grid-search was employed from the Scikit-learn toolkit for classification. Similarly, the specific steps of the algorithm were as follows: (1) Some hyperparameters are set as candidate values; (2) each hyperparameter combination is utilized to build an independent SVM model; and (3) the model and hyperparameter setting that produces the best results are selected, and three kernel functions are employed to set hyperparameters, including linear kernel function, polynomial kernel function, and radial basis kernel function.

In the classification step, the method of hyperparameter optimization was used to obtain better prediction effects and classification model performance. The grid search was used to adjust the parameters of SVM, and the 10-fold cross-validation was performed to avoid overfitting. The candidate values of the hyperparameters were set as the following 22 commonly used groups: linear (C = 1, 10, 100, 1,000), poly (C = 1, degree = 2, 3), or rbf (C = 1, 10, 100, 1,000, gamma = 1 or 0.1, 0.01, 0.001). As 10-fold validation could subdivide samples randomly, we repeated it 50 times in order to estimate the average and more reliable accuracy.

In fALFF analysis, the extracted features were ranked and weighted by RFECV with 10-fold cross-validation. After that, The N (1 ≤ *N* ≤ *extracted features*) ROIs with the highest weight were selected as features for classification by using the SVM optimized by 10-fold cross-validation grid-search. The features were investigated when the classification framework was applied to a specific frequency band or if the sliding window-width performed best. In fALFF analysis, the selected features with the best classification performance were the abnormal ROIs. The same cross-validation scheme for the dFC analysis was followed as for the fALFF analysis. In dFC analysis, the selected features with the best classification performance were the time-varying correlation characteristics for schizophrenia, which were thought to be the biomarkers for schizophrenia in this study.

#### 2.4.7. Model evaluation

Table 1 shows a contingency table of the types of errors and successes in classification for the presence of some anomaly, in general for the output of a binary classifier. Prediction can also be stated in general as “positive/negative.” The performance of classification was evaluated using accuracy (ACC), precision, recall, f1-score, receiver operating characteristic (ROC) curve, and area under the ROC curve (AUC).

**Table 1:** The definition of TP, TN, FP, and FN in classification.

**Case**	**Prediction**	**Reality (truth)**
False positive (FP)	Anomaly	Normal
False negative (FN)	Normal	Anomaly
True positive (TP)	Anomaly	Anomaly
True negative (TN)	Normal	Normal

## 3. Results and analysis

### 3.1. Fractional amplitude of results of low-frequency fluctuations

The initial fALFF feature set was 92 (46 + 46) × 90 (ROIs) per frequency band. Then, the RFECV with 10-fold cross-validation was performed to rank and weigh the ROIs in the Conventional scheme according to their strength to differentiate between the two groups. Similarly, the ROIs in the Slow-4, Slow-5, and Combined schemes were ranked and weighted.

**Figure 3:**
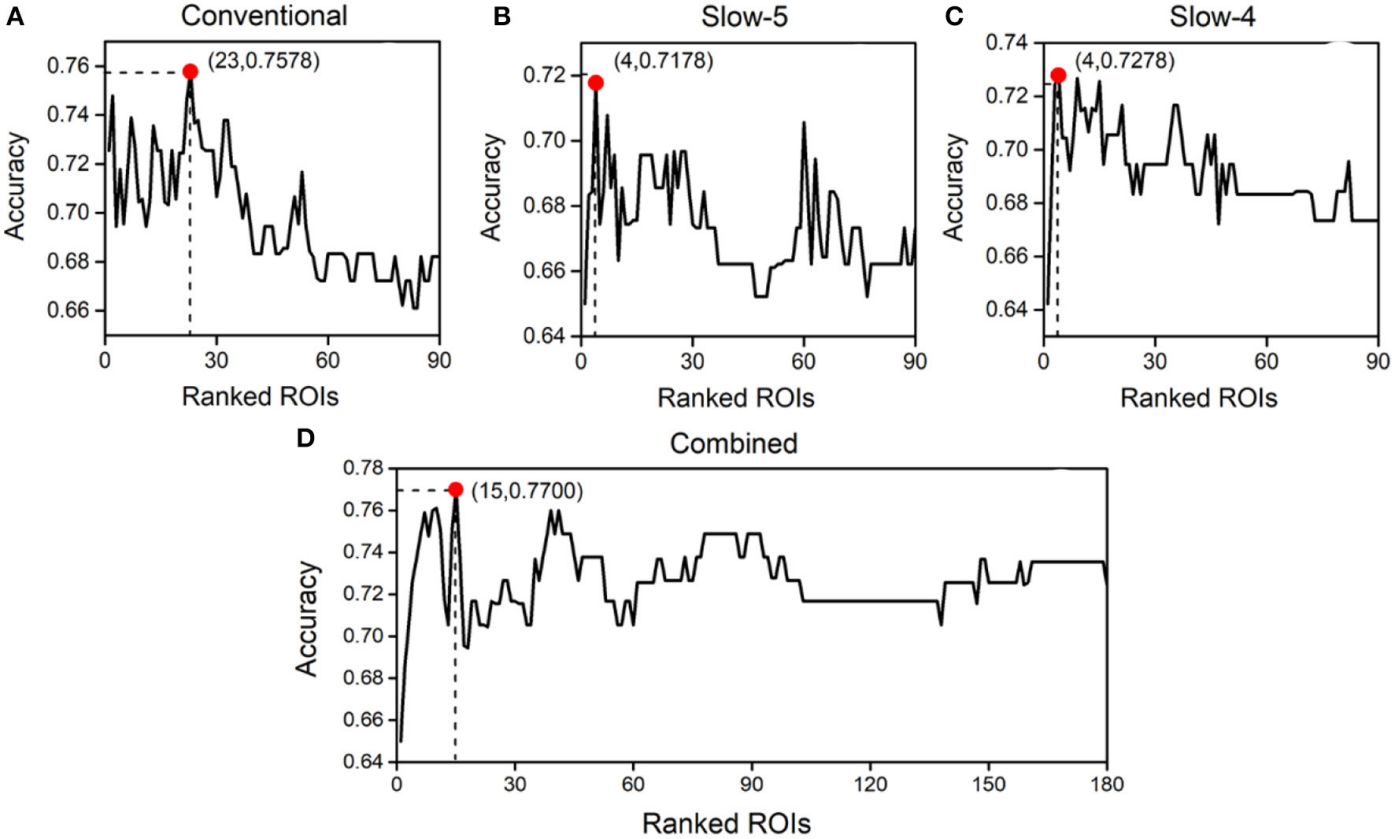
Classification accuracy obtained using different numbers of abnormal brain regions ranked by importance in the Conventional **(A)**, Slow-5 **(B)**, Slow-4 **(C)**, and Combined **(D)** schemes.

Thereafter, the SVM optimized by a 10-fold cross-validation grid search was applied to the feature set for identifying abnormal ROIs. Figure 3 depicts the classification accuracy of using the N (1 ≤ *N* ≤ *ranked features*) ROIs with the highest weight in the Conventional, Slow-5, Slow-4, and Combined schemes, respectively. The horizontal axis represents the number of top-ranked abnormal ROIs ranked for classification and the vertical coordinates represent the corresponding classification accuracy. The results showed that the highest classification accuracy in the Conventional scheme was 75.78% and the top-ranked 23 ROIs were abnormal. The 23 ROIs (ranked) were as follows: Occipital_Mid_R, Occipital_Inf_R, Calcarine_L, Cuneus_L, Parietal_Sup_R, Frontal_Mid_Orb_L, Parietal_Inf_R, Occipital_Sup_R, Temporal_Pole_Mid_L, Calcarine_R, Postcentral_R, Lingual_L, Frontal_Inf_Tri_L, Occipital_Mid_L, Postcentral_L, Angular_R, Frontal_Mid_Orb_R, Occipital_Inf_L, Paracentral_Lobule_L, Temporal_Pole_Sup_R, Lingual_R, Occipital_Sup_L, and Putamen_L. Furthermore, the highest classification accuracy was 71.78% in Slow-5 and 72.78% in Slow-4. This indicated that neither Slow-5 nor Slow-4 contained enough pathological information for schizophrenia identification. The highest classification accuracy in the Combined scheme was 77.00%, and the top-ranked 15 ROIs were abnormal. The 15 ROIs (ranked) were as follows: Frontal_Mid_Orb_L(Slow-5), Occipital_Mid_R (Slow-4), Occipital_Inf_R(Slow-5), Parietal_Sup_R(Slow-4), Calcarine_L(Slow-4), Parietal_Sup_L(Slow-4), Cuneus_L(Slow-4), Frontal_Mid_Orb_R(Slow-5), Occipital_Sup_L(Slow-4), Parietal_Inf_R(Slow-4), Calcarine_R(Slow-4), Lingual_L (Slow-4), Occipital_Inf_R(Slow-4), Rectus_R(Slow-5), and Cingulum_Ant_L(Slow-5). The names, important coefficients, and other information about the abnormal ROIs are portrayed in Table 2. An interesting observation is that Occipital_Inf_R was the abnormal cortical ROI in both Slow-5 and Slow-4 for schizophrenia identification.

**Table 2:** The abnormal ROIs of schizophrenia in the distributed frequency bands.

**Band**	**ROI**	**Weight**	**Band**	**ROI**	**Weight**
Conventional	Occipital_Mid_R	0.5997	Slow-5	Frontal_Mid_Orb_L	0.5322
Conventional	Occipital_Inf_R	0.5840	Slow-4	Occipital_Mid_R	0.4904
Conventional	Calcarine_L	0.5114	Slow-5	Occipital_Inf_R	0.4483
Conventional	Cuneus_L	0.4804	Slow-4	Parietal_Sup_R	0.4412
Conventional	Parietal_Sup_R	0.4431	Slow-4	Calcarine_L	0.4359
Conventional	Frontal_Mid_Orb_L	0.3927	Slow-4	Parietal_Sup_L	0.4244
Conventional	Parietal_Inf_R	0.3766	Slow-4	Cuneus_L	0.4096
Conventional	Occipital_Sup_R	0.3660	Slow-5	Frontal_Mid_Orb_R	0.3950
Conventional	Temporal_Pole_Mid_L	0.3609	Slow-4	Occipital_Sup_L	0.3760
Conventional	Calcarine_R	0.3420	Slow-4	Parietal_Inf_R	0.3732
Conventional	Postcentral_R	0.3037	Slow-4	Calcarine_R	0.3418
Conventional	Lingual_L	0.3002	Slow-4	Lingual_L	0.3314
Conventional	Frontal_Inf_Tri_L	0.2999	Slow-4	Occipital_Inf_R	0.3107
Conventional	Occipital_Mid_L	0.2911	Slow-5	Rectus_R	0.3087
Conventional	Postcentral_L	0.2902	Slow-5	Cingulum_Ant_L	0.3082
Conventional	Angular_R	0.2901			
Conventional	Frontal_Mid_Orb_R	0.2829			
Conventional	Occipital_Inf_L	0.2791			
Conventional	Paracentral_Lobule_L	0.2719			
Conventional	Temporal_Pole_Sup_R	0.2705			
Conventional	Lingual_R	0.2659			
Conventional	Occipital_Sup_L	0.2624			
Conventional	Putamen_L	0.2566			

**Figure 4:**
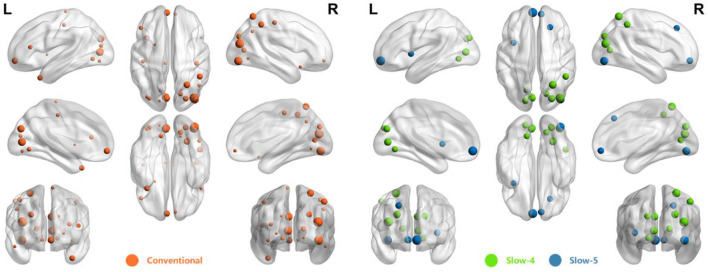
The distribution of abnormal ROIs obtained from Conventional and Combined schemes.

The abnormal ROIs in the Conventional and Combined schemes are mapped in Figure 4 using BrainNet Viewer (Xia et al., 2013). Particularly, a larger radius of nodes indicated higher importance. For the ROIs in both Slow-4 and Slow-5, only the one with higher importance coefficient was mapped. In fact, there were 23 ROIs in the Conventional and 15 ROIs in the Combined schemes. Specifically, there were five abnormal ROIs in Slow-5 and 10 abnormal ROIs in Slow-4. It can be seen that the extracted ROIs in the Combined scheme were similar to the ones in the Conventional scheme. Moreover, most of the extracted ROIs with high-importance coefficients in the Combined scheme also enjoyed a high ranking in the Conventional scheme.

### 3.2. Dynamic functional connectivity results

**Figure 5:**
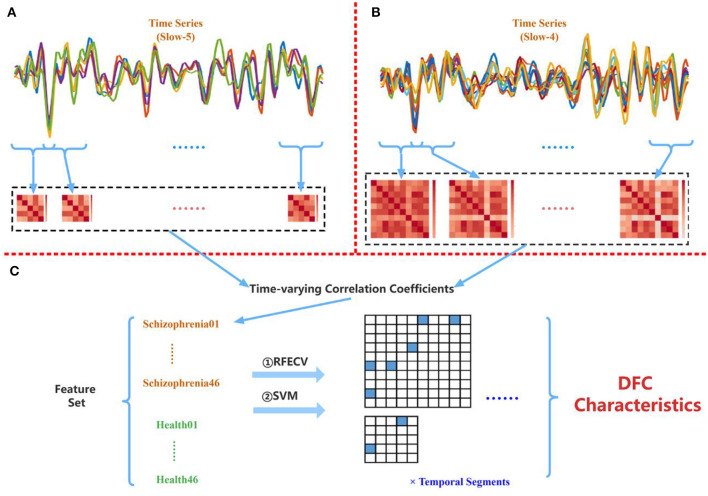
**(A)** Extracting PCC among five abnormal ROIs found in Slow-5. **(B)** Extracting PCC among 10 abnormal ROIs found in Slow-4. **(C)** Applying the proposed multi-frequency-based dFC method to the combined time-varying correlation coefficients.

Table 3 shows the frequency band scheme, the searching area of the width of the sliding window, the step length, the number of temporal segments per case, and the number of features per case. Two frequency band schemes (Conventional and Combined) were considered and four sliding window-widths (15 TRs, 20 TRs, 25 TRs, and 30 TRs) were analyzed. Figure 5 is the schematic diagram for investigating time-varying FC characteristics. For the Combined scheme, the processed time series of five targeted ROIs in Slow-5 and 10 targeted ROIs in Slow-4 were extracted from both the schizophrenia and healthy groups. For the analysis in the Conventional scheme, the five targeted ROIs in Slow-5 and 10 targeted ROIs in Slow-4 were utilized to extract their time series in the Conventional scheme. Then, the time-varying correlation coefficients among abnormal ROIs were calculated at each sliding window-width with five TRs step length. Thereafter, the two matrices (10 × 10, 5 × 5) obtained from each of the temporal segments were concatenated as the features for each subject. Particularly, each of the 92 subjects has 55 [10 × (10 – 1)/2 + 5 × (5 – 1)/2] correlation features at each temporal segment because the PCC matrices are symmetric.

**Table 3:** The frequency band scheme, sliding window-width, temporal segments, and number of dFC characteristics in each step of the feature selection per case.

**Frequency band scheme**	**Sliding window-width**	**Temporal segments**	**Feature set**	**dFC characteristics**
Combined	15 TRs	26	92 × 1,430	41
Combined	20 TRs	25	92 × 1,375	42
Combined	25 TRs	24	92 × 1,320	31
Combined	30 TRs	23	92 × 1,265	23
Conventional	15 TRs	26	92 × 1,430	33
Conventional	20 TRs	25	92 × 1,375	35
Conventional	25 TRs	24	92 × 1,320	30
Conventional	30 TRs	23	92 × 1,265	28

**Figure 6:**
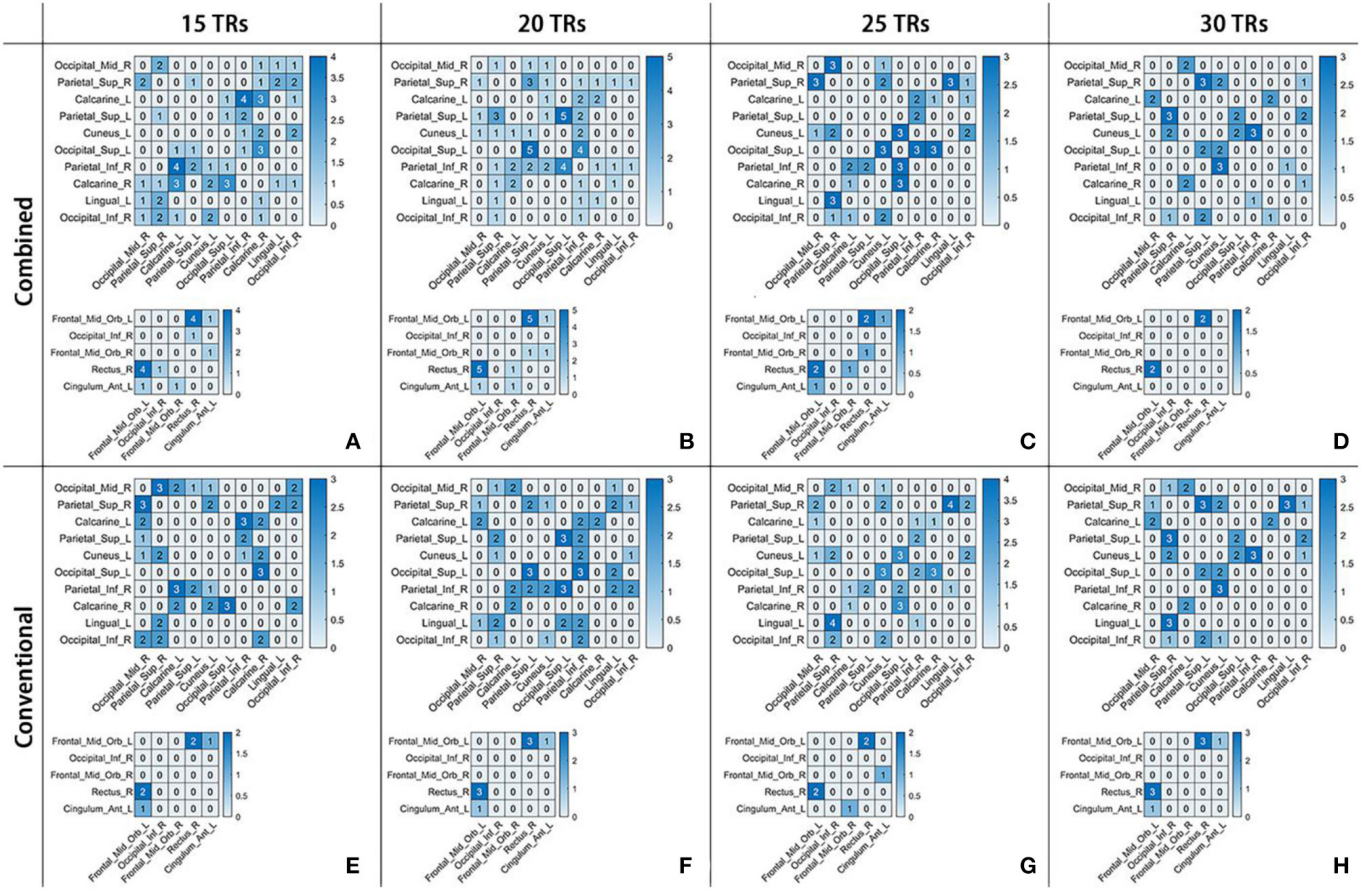
The number of time-varying correlation characteristics for schizophrenia in two frequency schemes at four sliding window-widths, as showed in **(A-D)** for Combined and **(E-H)** for Conventional. In each sub-figure, there were 10 × 10 and 5 × 5 matrices. The number in each grid represents the amounts of temporal segments with discriminative PCC in a certain frequency band scheme at a certain sliding window-width.

The specific number of time-varying correlation characteristics in each pair of abnormal ROIs per case is shown in Figure 6. In each grid, a number was presented to show the amounts of temporal segments with discriminative FC PCC between a specific pair of ROIs. This indicated that not all pairs of abnormal ROIs had correlation characteristics for schizophrenia. Moreover, the discriminative time-varying correlation coefficients were not exactly the same at the different temporal segments of the same sliding window-width.

Table 4 shows the classification results. In the Combined scheme, ACC and AUC were the highest at 20 TRs sliding window-width (91.89%, 0.9250). In the Conventional scheme, ACC and AUC were also the highest at 20 TRs sliding window-width (88.67%, 0.9205). It should be noticed that the highest ACC in the Conventional (88.67%) was lower than the highest ACC in the Combined (91.89%) scheme. In addition, the ACC in the Combined scheme was almost identical to that in the Conventional scheme at 25 TRs or 30 TRs sliding window-width, indicating that there was no significant difference between classification performance in both schemes. For the analysis on a certain sliding window-width, the important coefficients obtained from the time-varying correlation coefficients between the same pair of abnormal ROIs were similar in different cases of window-widths. This phenomenon indicated that the interaction of the human brain regions was dynamic as well as relatively stable. Therefore, our results supported the hypothesis that the multi-frequency-based dFC analysis method could provide more neuropathological information than the conventional band.

**Table 4:** The classification performance of different frequency band schemes in the cases of four sliding window-widths.

**Frequency band scheme**	**Sliding window-width**	**AUC**	**ACC**	**f1**	**Precision**	**Recall**
Combined	15 TRs	0.9222	91.21%	88.93%	89.29%	88.63%
Combined	20 TRs	0.9250	91.89%	89.49%	88.91%	90.16%
Combined	25 TRs	0.9052	85.91%	84.81%	85.43%	86.34%
Combined	30 TRs	0.8907	84.72%	82.35%	83.15%	84.27%
Conventional	15 TRs	0.9170	88.11%	86.10%	86.96%	86.20%
Conventional	20 TRs	0.9205	88.67%	84.35%	86.52%	89.57%
Conventional	25 TRs	0.8964	83.44%	84.26%	85.98%	85.87%
Conventional	30 TRs	0.8910	82.93%	82.58%	83.59%	85.43%

Furthermore, we also proposed a hypothesis that combing multiple frequency bands at distributed sliding window-widths may also be worthy of consideration, instead of the same sliding window-width. We carried out further analysis at 15 TRs and 20 TRs sliding window-widths because of their better performance in the Combined scheme. First, sliding window-width in Slow-5 was selected as 15 TRs while that of Slow-4 was selected as 20 TRs. Then, feature selection and classification methods were performed. Finally, the results of AUC and ACC were 0.9117 and 89.30%, respectively. Similarly, the sliding window-width of Slow-5 was selected as 15 TRs while that of Slow-4 was selected as 20 TRs. In this turn, both the AUC and ACC were 0.9154 and 90.07%, respectively. Table 5 indicates that it may also be meaningful to combine multiple frequency bands at distributed sliding window-widths.

**Table 5:** The classification performance in Slow-5 and Slow-4 at distributed sliding window-widths.

**Frequency band scheme**	**AUC**	**ACC**	**f1**	**Precision**	**Recall**
Slow-5 (15 TRs)	0.9222	91.21%	88.93%	89.29%	88.63%
Slow-4 (15 TRs)					
Slow-5 (20 TRs)	0.9250	91.89%	89.49%	88.91%	90.16%
Slow-4 (20 TRs)					
Slow-5 (15 TRs)	0.9117	89.30%	86.36%	88.23%	86.24%
Slow-4 (20 TRs)					
Slow-5 (20 TRs)	0.9154	90.07%	89.45%	90.52%	89.60%
Slow-4 (15 TRs)					

## 4. Discussion

Previous rs-fMRI studies for identifying abnormal ROIs of schizophrenia mostly focused on the Conventional scheme. For example, Bluhm et al. (2007) found abnormalities in the default network, Huang et al. found abnormalities in the frontal lobe and occipital lobe in patients with schizophrenia (Huang et al., 2010), and Scheinost et al. (2019) also found a similar set of regions with schizophrenia and controls. These findings are consistent with our findings that the Frontal_Mid_Orb_L, Frontal_Inf_Tri_L, Frontal_Mid_Orb_R, Occipital_Mid_R, Occipital_Inf_R, Occipital_Mid_L, Occipital_Inf_L, and Occipital_Sup_L were the abnormal brain regions in the Conventional scheme, while there were few rs-fMRI studies based on the method of multi-frequency bands. Meanwhile, most of these studies concentrated on exploring the neuropathological mechanism of schizophrenia from a frequency-specific perspective. For instance, Yu et al. (2014) found that there were significant distinctions in the basal ganglia, midbrain, and ventromedial prefrontal cortex between Slow-4 and Slow-5. They also found that there were distinctions in interactions among the inferior occipital gyrus, precuneus, and thalamus. Moreover, the significant interaction effects between frequency and group were observed in the left calcarine cortex, bilateral inferior orbitofrontal gyrus, and anterior cingulum cortex (Luo et al., 2020) in Slow-5 and Slow-4. These results demonstrated that the abnormalities of brain regions and dFCs in schizophrenia patients rely on different frequency bands.

Furthermore, there are also some multi-frequency scheme researches from a feature fusion view. For example, Huang et al. (2018) acquired fALFF data in multi-frequency bands and combined them for classification. The biomarkers from Slow-4 and Slow-5 could achieve a classification accuracy of 85.3% on 34 subjects. These abnormal ROIs were consistent with our findings: Frontal_Mid_Orb_L (Slow-5), Cingulum_Ant_L (Slow-5), Cuneus_L (Slow-4), Calcarine_L (Slow-4), and Occipital_Sup_R (Slow-4). Wang et al. (2019) also demonstrated that the multi-frequency scheme shows promising performance in brain disease classification. These abnormal ROIs were consistent with our findings: Cingulum.Ant.L (Slow-5), Occipital.Mid.R (Slow-4), and Parietal.Inf.L (Slow-4).

In recent years, an increasing number of studies have used dFC analysis to explore changes in neural activity patterns of schizophrenia (Nomi et al., 2016; Liu et al., 2017). A multi-frequency-based dFC analysis may further provide more evidence to explore the pathogeny of schizophrenia (Luo et al., 2020). For example, Zou and Yang (2019) performed their proposed dynamic-weighted FC networks to 130 subjects and obtained a classification accuracy of 82.31%. In this study, the first proposed multi-frequency-based dFC analysis could obtain a better classification performance than the Conventional scheme in some cases. For example, ACC and AUC were the highest at 20 TRs sliding window-width (86.89%, 0.9150). For the time-varying correlation characteristics, the interaction between Frontal_Mid_Orb_L and Rectus_R in Slow-5 was discriminative at four kinds of sliding window-width. Furthermore, in Slow-4, the interaction between Calcanie_L and Parietal_Inf_R at 15 TRs, and the interaction between Parietal_Inf_R and Occipital_Sup_L at 20 TRs and many interactions of other pairs of abnormal ROIs were discriminative. Some of these ROIs are included in the default mode network and cortical areas, which were the regions that found a remarkable difference between the two bands (Slow-4 and Slow-5) (Wang et al., 2017).

In this study, some abnormal ROIs found in the Conventional scheme were the same as the ones in the Combined scheme, as well as their rank and weight according to their importance coefficients. For example, Occipital_Mid_R, Occipital_Inf_R, Calcarine_L, Cuneus_L, and Parietal_Sup_R were abnormal and ranked highest in both Conventional and Combined schemes. This phenomenon is similar to previous studies (Huang et al., 2018; Wang et al., 2019; Tian et al., 2020). It reflected that the neuronal activities within each of the ROIs were variable but did not change significantly in dierent frequency bands (Fox et al., 2005; Sadaghiani and Kleinschmidt, 2013). For the importance coefficients of the time-varying correlation coefficients between the same pair of the abnormal ROIs, they were similar at some temporal segments of a certain sliding window-width but not at each temporal segment. This indicated that the interaction of the neuronal activities was dynamic but relatively stable. These phenomena proved that our experiments were reasonable and revealed some neuropathological mechanisms of schizophrenia.

Step length and sliding window-width are the key parameters affecting the SWC technique, and their selection will have a significant impact on the results of the dFC analysis. Therefore, the statistical comparison between DFCs corresponding to different frequency bands with four different window-widths was considered in the study. Considering the fact that there are significant differences in DFC between different frequency bands with different window-widths, this study mainly analyzes the case of the step length with 1TR, which was used in many studies, while the influence of different step lengths on the results of this study will be further investigated when more data are obtained in future. Furthermore, as a classical blind source separation method, independent component analysis has a good performance in the detection of abnormal brain functional areas, thus, the DFC influence between brain functional networks in different frequency bands on SZ also needs to be further explored based on this study in future.

## 5. Conclusion

In this study, we proposed a novel multi-frequency-based dynamic FC analysis for schizophrenia using resting-state fMRI data, which involves two parts: feature selection and classification for identifying abnormalities in ROIs. The same scheme was applied to the interactions of the abnormal ROIs for investigating time-varying characteristics. Experiments from 92 subjects demonstrated that the multi-frequency schemes had a promising performance in revealing meaningful disease-related biomarkers for schizophrenia. The experimental results suggested the performance of combining multiple features from different frequency bands is better than the conventional scheme in some cases: Slow-4 may contain additional useful information compared to Slow-5, and the combined scheme may provide more potential implications than the conventional scheme. Significantly, the abnormal brain regions in Slow-5 were Frontal_Mid_Orb_L, Occipital_Inf_R, Frontal_Mid_Orb_R, Rectus_R, and Cingulum_Ant_L, and the abnormal brain regions in Slow-4 were Occipital_Mid_R, Parietal_Sup_R, Calcarine_L, Parietal_Sup_L, Cuneus_L, Occipital_Sup_L, Parietal_Inf_R, Calcarine_R, Lingual_L, and Occipital_Inf_R. Moreover, the time-varying correlation characteristics for schizophrenia were also investigated in a multi-frequency scheme, which may provide objective and quantitative biomarkers for schizophrenia and valuable references for auxiliary psychiatric diagnosis.

## Data availability statement

Publicly available datasets were analyzed in this study. This data can be found at: https://openfmri.org/dataset/ds000030/.

## Ethics statement

All the data was collected from the public database OpenfMRI (https://openfmri.org/dataset/ds000030/). After receiving a thorough explanation, participants gave written informed consent following procedures approved by the Institutional Review Boards at UCLA and the Los Angeles County Department of Mental Health.

## Author contributions

ZS and YS: design of the study. ZS, YS, and LZ: analysis and interpretation. ZS and SL: drafting the article. YS, WZ, and NW: review article. All authors contributed to the article and approved the submitted version.
